# Impact on PET spatial resolution through positron range confinement in a high magnetic field across tissue-equivalent materials

**DOI:** 10.1088/1361-6560/ae6d7c

**Published:** 2026-05-26

**Authors:** F Lopez-Berenguer, A Gonzalez-Montoro, M Freire, S S Berr, M B Williams, A J González

**Affiliations:** 1 Instituto de Instrumentación para Imagen Molecular (I3M), Centro Mixto CSIC—Universitat Politècnica de València, Camino de Vera s/n, Valencia 46022, Spain; 2 The University of Virginia, Charlottesville, VA 22903, United States of America

**Keywords:** positron range, spatial resolution, preclinical PET imaging, PET/MRI, high magnetic field

## Abstract

O*bjective.* The spatial resolution of positron emission tomography (PET) imaging is intrinsically limited by the finite range of positrons before annihilation, an effect that becomes increasingly relevant for high-energy emitters and for preclinical studies targeting small structures. In integrated PET/ magnetic resonance imaging (MRI) systems, the magnetic field can modify positron trajectories, reducing their transverse spread and introducing anisotropic resolution effects. However, experimental evidence of the combined influence of positron energy and material density under high-field preclinical conditions remains limited. This work experimentally investigates the effect of a 9.4 T magnetic field on positron-range blurring for different radionuclides and tissue-equivalent phantoms in a preclinical PET/MRI environment. *Approach.* Experiments were performed using a high-resolution preclinical PET insert operating simultaneously inside a preclinical 9.4 T MRI system to study three positron-emitting radionuclides (^18^F, ⁸⁹Zr and ^6^⁸Ga). Capillary line sources were embedded in three tissue-equivalent phantoms with increasing density. Spatial resolution was quantified along the three spatial directions using the full width at half maximum and full width at tenth maximum (FWTM). Complementary measurements with a microDerenzo phantom were performed to assess rod resolvability. *Main results.* In low-density material, spatial resolution remains essentially unchanged by the magnetic field for all radionuclides. In contrast, in medium- and high-density materials, a marked transverse confinement is observed at 9.4 T for ^6^⁸Ga, with transverse FWTM values reduced by about 60% compared with 0 T, while the axial component remains largely unaffected. For ⁸⁹Zr, transverse improvements of about 25% are observed. These trends are consistent with the microDerenzo results: for ^6^⁸Ga, no rod sector is resolvable at 0 T, whereas at 9.4 T rods close to 1.0 mm become resolvable. *Significance.* This study provides a systematic experimental assessment of positron-range confinement in high-field preclinical PET/MRI and demonstrates that the magnitude of magnetic-field-induced resolution improvements depends on both positron energy and material density.

## 1. Introduction

The integration of positron emission tomography (PET) and magnetic resonance imaging (MRI) has received significant attention in the last decades, leading to the development of hybrid PET/MRI systems both in the clinical and preclinical fields (Judenhofer and Cherry 2013). This multimodal approach offers several advantages when compared to the standardized PET/computed tomography (CT) approach. PET/MRI has superior soft tissue contrast, reduced exposure to ionizing radiation, and the ability to simultaneously combine functional PET data with high-resolution anatomical and physiological information from MRI (Ehman *et al* 2017). These benefits have driven the adoption of PET/MRI in the clinical practice, particularly in fields such as oncology, neurology, and cardiology, where soft tissue contrast and functional imaging are crucial (Chen *et al* 2005, Herzog and Van Den Hoff 2012, Mannheim *et al* 2018, Nensa *et al* 2018). In preclinical studies, PET/MRI is of particular interest because the small size of biological structures makes image resolution and quantification especially challenging, as for instance in advanced research of neuroinflammation, tumor biology and metabolic disorders (James and Gambhir 2012, Ory *et al* 2015), among others.

The performance of PET systems is influenced by several factors, such as detector design and geometry, electronics, photon attenuation, partial volume effects, and patient motion (Daou 2008, Hofmann *et al* 2008, Bettinardi *et al* 2014, González *et al* 2016). Among these, one fundamental physical limitation that is independent of the detector technology is the positron range, which is the distance this particle travels before it annihilates with an electron of the surrounding medium (Moses 2011). The random motion of the positron traversing tissues introduces uncertainty in the localization of the radiotracer, since it results in a random spatial offset between the location of the decaying isotope atom and the point of positron-electron annihilation. This uncertainty then affects the lines of response, consequently blurring the reconstructed image (Phelps *et al* 1975). The extent of this effect depends on the initial kinetic energy of the positron and the density of the surrounding medium. While low-energy positron emitters such as fluorine-18 (^18^F) result in a short positron range contribution, high-energy emitters like zirconium-89 (⁸⁹Zr), gallium-68 (^6^⁸Ga) or rubidium-82 (^82^Rb) exhibit longer ranges and more pronounced effects (Van Dalen *et al* 2008). This is particularly critical in preclinical imaging, where the goal is to resolve small structures.

In PET/MRI systems, the static uniform magnetic field (*B*_0_) has field lines oriented parallel to the long axis of the magnet. The magnetic component of the Lorenz force, which affects charged particles moving in magnetic fields, acts perpendicularly to both *B*_0_ and the positron velocity. It thus produces no change in the positron’s axial velocity, but results in circular positron trajectories in the transverse plane. The result is a reduction of the effective transverse blurring, while the axial positron range remains essentially unaffected (Huang *et al* 2014). This concept dates to the early development of integrated PET/MRI systems, where initial designs already considered the possibility of using the magnetic field to reduce the transverse spread of positrons (Hammer *et al* 1994, Shao *et al* 1997). This effect becomes increasingly relevant for high-energy positron emitters and for strong magnetic fields, as are typically available in preclinical MRI systems (Wirrwar *et al* 1997).

Monte Carlo studies and experimental investigations have consistently demonstrated a reduction of the transverse positron range in magnetic fields of 3 T and above, with a stronger effect for high-energy positron emitters such as ^6^⁸Ga (Soultanidis *et al* 2011, Huang *et al* 2014, Li *et al* 2017). Similar effects have also been observed at higher magnetic fields, as shown by Shah *et al* (2014) using a PET system combined with a 9.4 T MRI scanner and a brain phantom, where medium- and high-energy emitters exhibited a significant improvement in spatial resolution.

From a translational perspective, the potential for magnetic reduction of positron range is particularly relevant for radiotracers such as ^6^⁸Ga and ⁸⁹Zr, which are widely used in oncology and immuno-PET studies. ^6^⁸Ga-labeled tracers are commonly employed in neuroinflammation imaging, tumor targeting and peptide-receptor imaging because of their favorable kinetics and generator availability (Cheng *et al* 2018, Li *et al* 2023, Luo *et al* 2025), whereas ⁸⁹Zr-labeled antibodies are increasingly used for longitudinal tracking of monoclonal antibodies in oncology and immunotherapy research (Dijkers *et al* 2010). Preclinical PET/MRI studies at high magnetic fields therefore provide a unique platform to investigate how positron range confinement affects image resolution and, ultimately, quantitative accuracy for these clinically relevant isotopes under well-controlled conditions.

The impact of the positron range is also strongly dependent on tissue density. Low-density media such as lung result in substantially longer positron paths than denser tissues due to their lower electron densities (Kraus *et al* 2012). Experimental studies using tissue-equivalent phantoms have shown that identical positron emitters produce significantly broader positron range distributions in low-density media, leading to increased blurring and underestimation of activity concentration (Alva-Sánchez *et al* 2016). More recently, a study performed in a clinical PET/MRI system at 3 T that the magnetic field significantly reduced the transverse extent of the ^6^⁸Ga positron range distribution in lung-equivalent media and improved recovery coefficients, without inducing axial elongation (Ku-Toval *et al* 2024), without inducing axial elongation.

In this work, we present an experimental study conducted in a 9.4 T preclinical MRI system using a custom high-resolution dedicated PET insert based on monolithic LYSO:Ce crystals. Our experimental data includes capillary-based tests and a microDerenzo phantom, both designed at geometric scales representative of mice structures. We tested positron emitters with three different positron emission energies (^18^F, ⁸⁹Zr, and ^6^⁸Ga) that were surrounded by tissue-equivalent materials of three different physical densities. Our goal was to experimentally characterize positron range confinement in a high-field MRI environment and assess how it influences PET image resolution.

Our findings contribute to the experimental characterization of positron behavior in high magnetic fields and to the assessment of positron range confinement effects on PET spatial resolution. The observed reduction of the effective transverse positron range is expected to mitigate partial volume effects in small structures and may therefore contribute to improved quantitative accuracy in preclinical PET studies (Soret *et al* 2007). This is particularly relevant in preclinical oncology and neuroimaging applications, such as small-animal brain tumor models, where typical lesion sizes are only a few millimeters and improvements in effective spatial resolution are expected to directly translate into reduced partial volume effect, better visualization of small structures, and more reliable activity estimation. A dedicated quantitative accuracy evaluation is left for future studies.

## 2. Materials and methods

### 2.1. PET/MRI system

The study was performed using a custom-built preclinical PET insert composed of 16 monolithic LYSO:Ce crystals arranged in a two-ring octagonal geometry with an inner diameter of 72 mm and an axial field-of-view (FOV) of 67 mm. Each scintillation crystal (33 × 25.4 × 8 mm^3^) provides accurate 3D interaction positioning with depth of interaction (DOI) capabilities, minimizing parallax errors and ensuring a uniform spatial resolution across the FOV (Lopez-Berenguer *et al* 2025). The PET insert performance was previously evaluated following the NEMA NU-4 protocol. A uniform spatial resolution of approximately 0.9 mm full width at half maximum (FWHM) was achieved when DOI information was considered. The system showed a peak sensitivity of 3.8% for a 255–766 keV energy window, a peak noise-equivalent count rate of 80 kcps at an activity of 19 MBq, and recovery coefficients (RCs) ranging from 0.31 to 0.89 for the 1–5 mm rods of the NEMA NU-4 image quality phantom, as reported in our previous work (Lopez-Berenguer *et al* 2025).

A Voronoi-based calibration procedure was applied to accurately determine the 3D interaction coordinates and the deposited energy (Freire *et al* 2019). The PET was designed to be inserted in a 9.4 T Bruker Biospec 94/20 MRI (Bruker Corporation 2024). To minimize electromagnetic coupling with the RF field, the PET insert was shielded using a three-layer carbon-fiber cage, which does not introduce measurable perturbations of the static magnetic field B_0_ during the experiments (Gonzalez *et al* 2018, Gsell *et al* 2020).

All PET measurements performed inside the MRI (B_0_ = 9.4 T) were acquired without running MR sequences during data acquisition, to isolate the effect of the static magnetic field on positron range.

### 2.2. Experimental measurements

Glass capillaries were used as line sources in one of the studies. Each capillary tube had inner and outer diameters of 1.15 mm and 1.5 mm, respectively, with ~39 mm in length, resulting in a fillable volume of 40 *µ*l. The capillaries were filled by capillary action with ^18^F, ⁸⁹Zr or ^6^⁸Ga solutions using activities in the range of 3.7–7.5 MBq. The activity of each capillary was measured using a dose calibrator and decay-corrected to the acquisition start time. For each measurement, the three radionuclides were positioned simultaneously in the phantom and acquired in a single 120 s PET scan.

**Figure 1.**
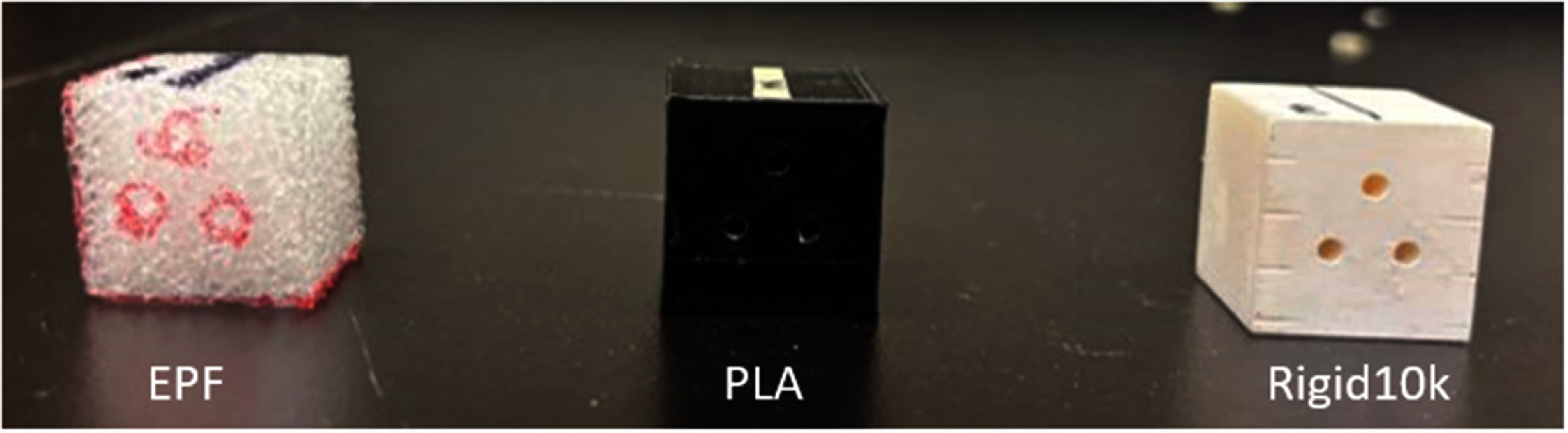
Photograph of the three custom capillary phantoms used in the study. From left to right: low-density EPF, medium-density PLA and high-density rigid10k resin.

To investigate the influence of the surrounding medium on positron-range blurring, three custom phantoms were fabricated from materials with different effective densities. Expanded polystyrene foam (EPF) (Finnfoam S.L 2025), polylactic acid (PLA) (RS PRO 2025), and Rigid10k resin (Formlabs 2022) were selected to provide low-, medium-, and high-density environments, respectively, as summarized in table 1. Each phantom consisted of a 25 × 25 × 25 mm^3^ cube containing three longitudinal holes designed to host the capillaries. They were positioned at the PET centered FOV for all experiments. The capillaries were arranged in the transverse plane forming an approximately equilateral triangle, enabling simultaneous acquisition under identical geometric and magnetic-field conditions. The three phantoms are shown in figure 1.

**Table 1.** Description of the tissue-equivalent characteristics of the three capillary phantoms used in this study.

	Material	Density (g cm^–3^)	Similar tissue
*Low-density*	EPF	0.20	Lung inhale
Medium-density	PLA	1.30	Trabecular bone
High-density	Rigid10k Resin	1.70	Cortical bone

The main properties of the three isotopes used in this study are summarized in table 2. These values have been obtained using the ICRP tissue data reported by Carter *et al* (2020) and following the density-scaling approach described by Cal-González *et al* (2013).

**Table 2.** Physical properties of the radionuclides used in the study and density-adjusted median positron ranges are estimated for the three phantom materials.

Isotope	Half-life (min)	${E_{\beta max}}$ (MeV)	${R_{median}}$ EPF (mm)	${R_{median}}$ PLA (mm)	${R_{median}}$ Rigid10K (mm)
^18^F	110	0.63	2.12	0.34	0.24
⁸⁹Zr	4704	0.90	4.31	0.70	0.49
^6^⁸Ga	68	1.90	12.02	1.97	1.37

**Figure 2.**
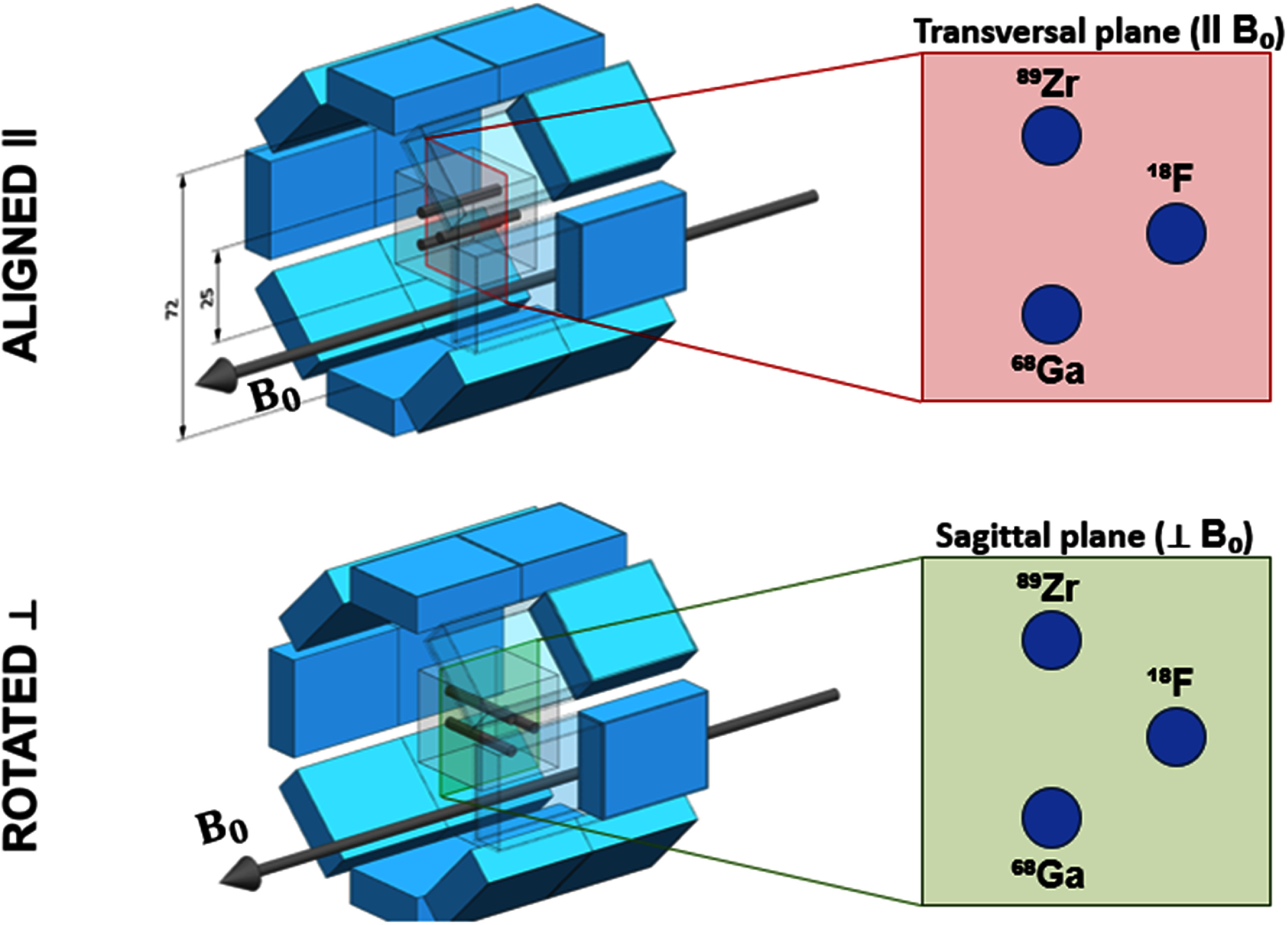
Schematic representation of the two phantom orientations used in the experiments. In the aligned (‖) configuration, the capillaries were oriented parallel to ${B_0}$. In the rotated (⊥) configuration, the phantom was rotated by 90°, placing the capillaries perpendicular to ${B_0}$. The dimensions of the phantom and the PET system are in mm. For visualization purposes, several detector modules are hidden in the schematic; the actual system consists of a complete detector ring.

For both outside-MRI (0 T) and inside-MRI (9.4 T) acquisitions, each phantom was imaged in two orthogonal orientations as shown in figure 2. In the aligned configuration, the capillaries were axially oriented with the PET axis, that is parallel to ${B_0}$ (labeled as ‖). In the rotated configuration, the phantom was rotated by 90°, placing the capillaries in an orientation perpendicular to the previous case (perpendicular to ${B_0}$, ┴). These orientations refer to the physical orientation of the capillaries with respect to the static magnetic field ${B_0}$. Using the capillaries as line sources, both orientations allowed us to study the performance of the three spatial directions (x, y and z) for all materials and isotopes and enabled direct comparison between measurements acquired outside the MRI and those acquired under the 9.4 T magnetic field.

In a second study, a microDerenzo phantom (Phantech LLC, model D270715-GrIT) was used to characterize the spatial resolution of the PET system. The phantom contains six radial sectors with rods of 0.7, 0.8, 0.9, 1.0, 1.2 and 1.5 mm in diameter (10 mm length), arranged within a cylindrical housing of 27 mm outer diameter. This configuration provides a well-defined pattern for assessing rod detectability at a submillimeter scale. The phantom was filled with ^18^F, ⁸⁹Zr and ^6^⁸Ga, using approximately 7.4 MBq of activity each (in different days and after validating no contamination from former isotopes), and each PET scan was acquired under identical condition for 1 h.

For all isotopes, acquisitions were performed both outside and inside the 9.4 T MRI system. For ^18^F and ⁸⁹Zr inside the MRI, only the orientation with rods parallel to the B_0_ field lines (||) was used, and the perpendicular orientation was not used. For ^6^⁸Ga, both parallel and perpendicular (┴) rod orientations were tested.

### 2.3. Image reconstruction

All datasets were reconstructed using a GPU-accelerated MLEM algorithm (Shepp and Vardi 1982) with 0.5 mm isotropic voxel size. An energy filter of 408–613 keV was applied to the list-mode data. This narrower window, compared to the 255–766 keV window used in the NEMA characterization of the system (Lopez-Berenguer *et al* 2025), was chosen to reduce the influence of scatter and random coincidences arising from the simultaneous acquisition of three radionuclides. No corrections for attenuation, scatter, random coincidences or dead time were applied.

For the capillary experiments, 10 MLEM iterations were used. This value was determined from an optimization study in which the FWHM and full width at tenth maximum (FWTM) were studied as a function of the iteration number for the three isotopes and the three phantom materials. Although high iterations produced slightly narrower FWHM values, they also broadened the FWTM, consistent with previous observation using a small-size source (Lopez-Berenguer *et al* 2025). For the microDerenzo phantom, 150 MLEM iterations were used, also following the reconstruction protocol established in our previous evaluation of this PET insert (Lopez-Berenguer *et al* 2025). The number of iterations was selected as it provides sufficient convergence for rod resolvability tasks, while avoiding the noise amplification associated with higher iteration counts.

### 2.4. Data analysis

To assess the spatial resolution along the three spatial directions, PET images were acquired for each of the two phantom orientations shown in figure 2. For the configuration with the rods aligned axially, the transverse plane (‖ *B*_0_) of the reconstructed images was used to extract the *x*- and *y*-direction profiles, while the *z*-direction profiles were obtained from the sagittal plane (⊥ *B*_0_). This scheme ensured that all three spatial directions were consistently sampled for each isotope and phantom material.

For each profile (see figure 4), the peak position was estimated by fitting a local parabola to the three profile points surrounding the maximum intensity (the highest point and its two nearest neighbors). The vertex of this parabola provided an estimation of the peak intensity and its location. The FWHM and FWTM were then calculated from the interpolated intensity profile at the half-maximum and tenth-maximum levels. To increase statistical robustness, the same analysis was repeated for the slice passing through the center of the capillary and for the three immediately adjacent slices above and below it. The resulting FWHM and FWTM values were then averaged to reduce slice-to-slice statistical fluctuations. The uncertainties are reported as the standard deviation across these seven measurements, which are small and primarily reflect the local parabolic fitting procedure used to estimate the peak position and intensity.

The rod resolvability in the microDerenzo phantom was assessed using the Rayleigh criterion (Hallen *et al* 2020, Toussaint and Loignon-Houle 2023). For each sector of the phantom, line profiles were extracted across the rod array, and the peak and valley intensities of every adjacent rod pair were obtained using local parabolic fits. The valley-to-peak ratio (VPR) was then computed for each pair. A sector was considered resolvable when the mean VPR computed for all rod pairs within that sector fell below the Rayleigh threshold of 0.735.

## 3. Results

### 3.1. Capillaries

**Figure 3.**
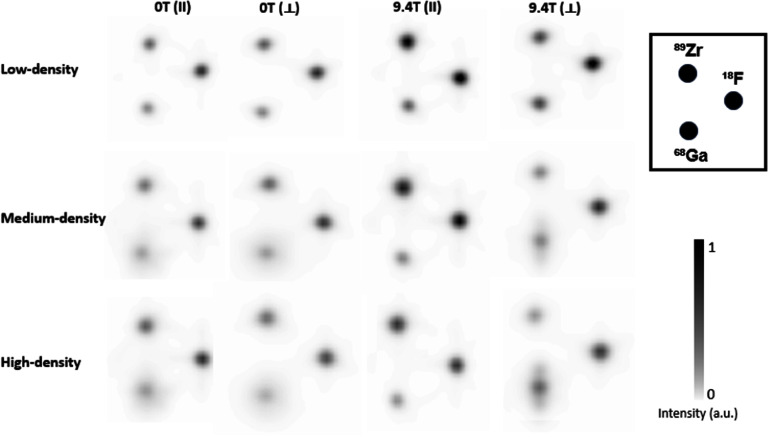
Reconstructed images of the three capillary phantoms (low-, medium-, and high-density) acquired outside the magnetic field (0 T) and inside (9.4 T) the MRI system. For each field condition, two views are shown: the transverse plane obtained in the aligned configuration (‖) and the sagittal plane obtained in the rotated configuration (⊥). All images are displayed using the same normalized intensity scale (color bar shown on the right).

figure 3 shows, for each capillary phantom, the reconstructed images acquired at 0 T and at 9.4 T. For each magnetic-field condition, two views are displayed: the transverse plane obtained in the aligned configuration (‖ *B*_0_) and the sagittal plane obtained in the rotated configuration (⊥ *B*_0_), as defined in figure 2. The three isotopes (^18^F, ⁸⁹Zr and ^6^⁸Ga) were imaged simultaneously.

**Figure 4.**
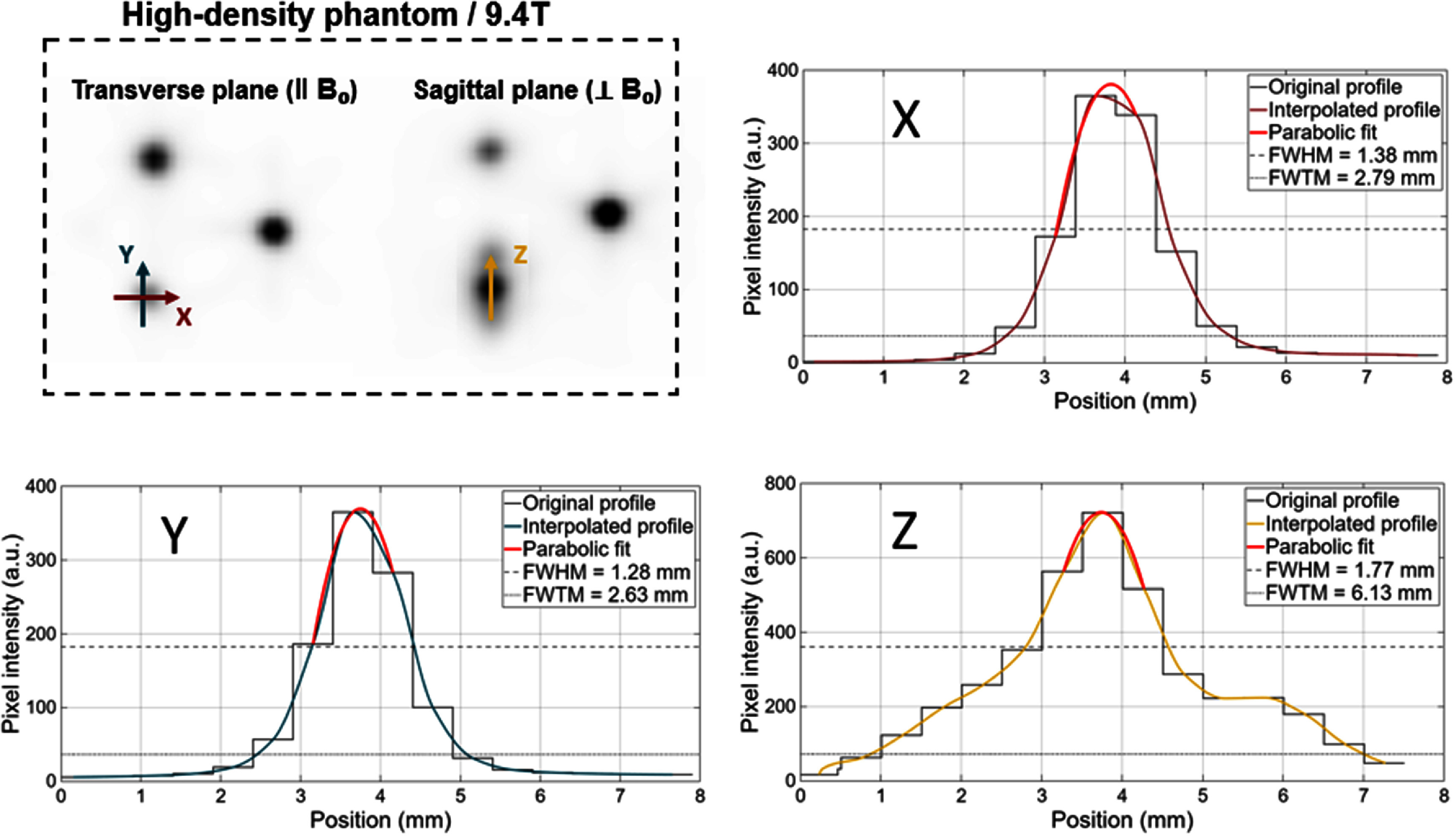
Example of the profiles extracted for high-density phantom imaged at 9.4 T with ^6^⁸Ga, showing the transverse (‖) and sagittal (⊥) reconstructions together with the corresponding intensity profiles along the x (red), y (blue) and z (yellow) directions.

An example of how the quantitative metrics were obtained for the ^6^⁸Ga capillary in the high-density phantom at 9.4 T is shown in figure 4. The transverse (‖ *B*_0_) and sagittal (⊥ *B*_0_) planes of the image reconstructions are shown together with the corresponding intensity profiles along the *x, y* and *z* directions, extracted following the procedure described in the Data Analysis section. The *x, y* and *z* profiles are displayed as solid red, blue. and yellow lines, respectively.

**Figure 5.**
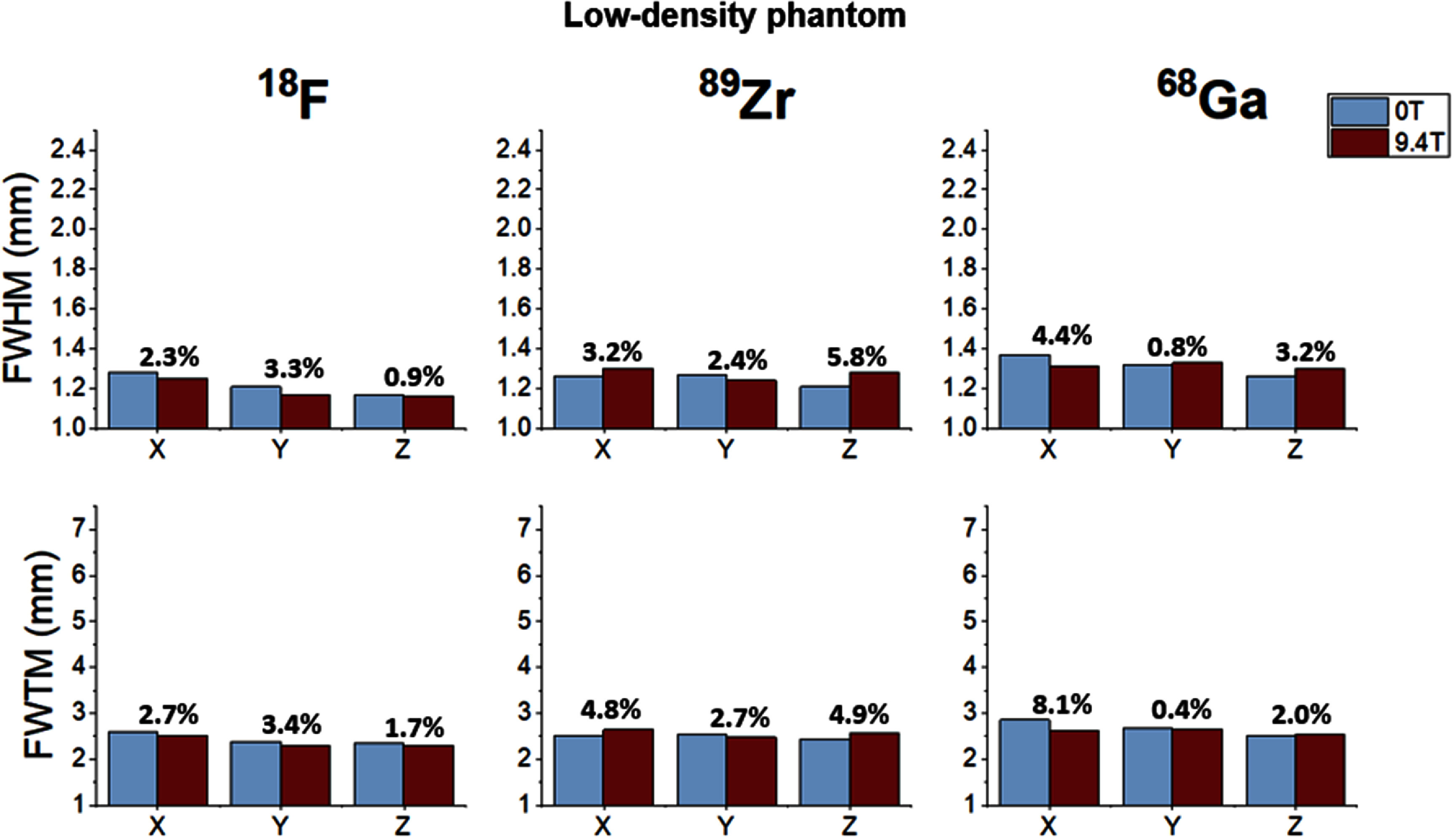
FWHM (top row) and FWTM (bottom row) obtained from the capillary profiles for low-density phantom. For each isotope (^18^F, ⁸⁹Zr, ^6^⁸Ga), the metrics are shown along the three spatial directions (x, y, z) and for the two MRI field conditions (0 T in blue and 9.4 T in red). The percentage numbers above the bars indicate the relative change between 9.4 T and 0 T. Error bars represent the standard deviation across the seven analyzed slices.

**Figure 6.**
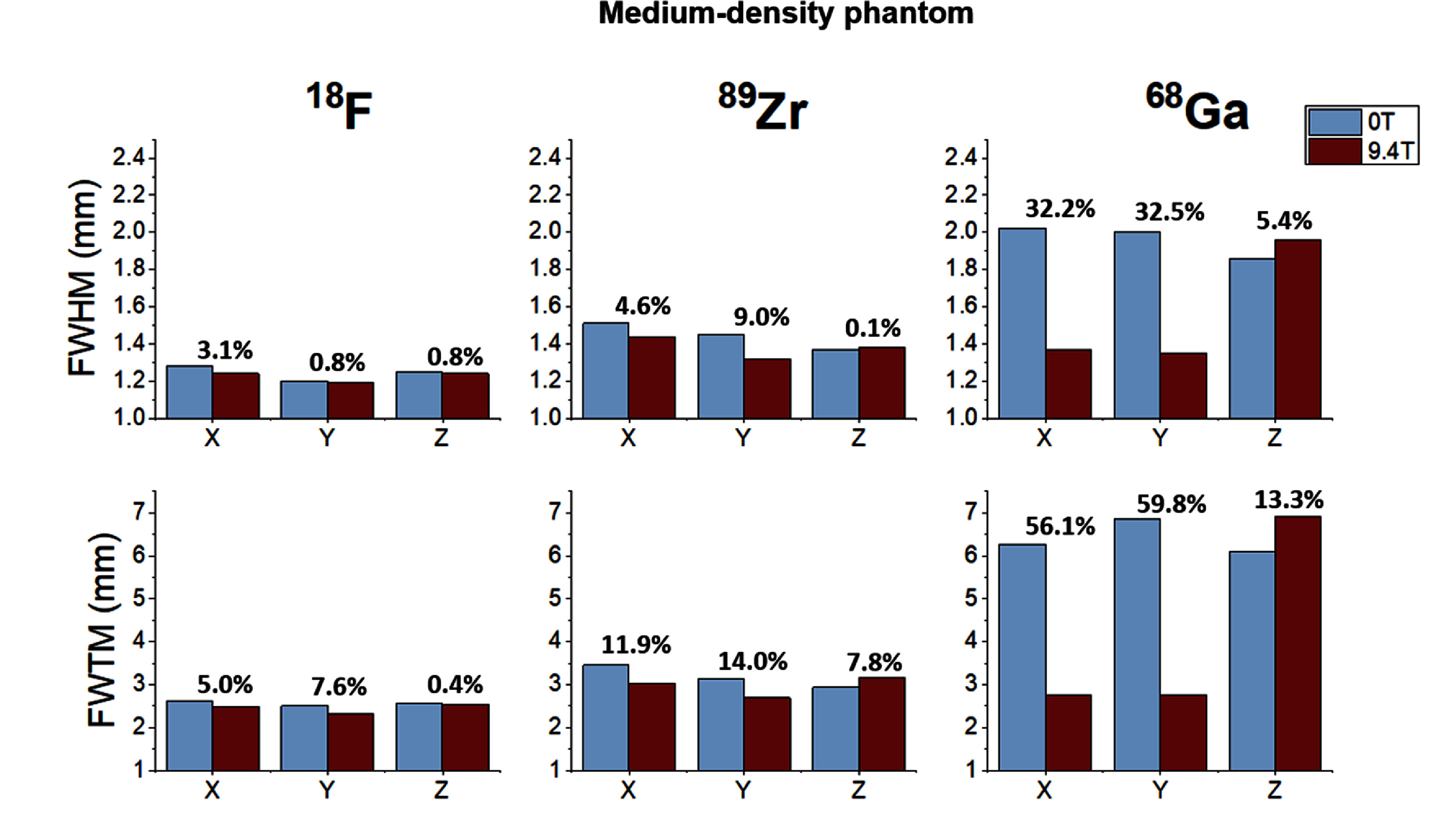
FWHM (top row) and FWTM (bottom row) obtained from the capillary profiles for medium-density phantom. For each isotope (^18^F, ⁸⁹Zr, ^6^⁸Ga), the metrics are shown along the three spatial directions (x, y, z) and for the two MRI field conditions (0 T in blue and 9.4 T in red). The percentage numbers above the bars indicate the relative change between 9.4 T and 0 T. Error bars represent the standard deviation across the seven analyzed slices.

**Figure 7.**
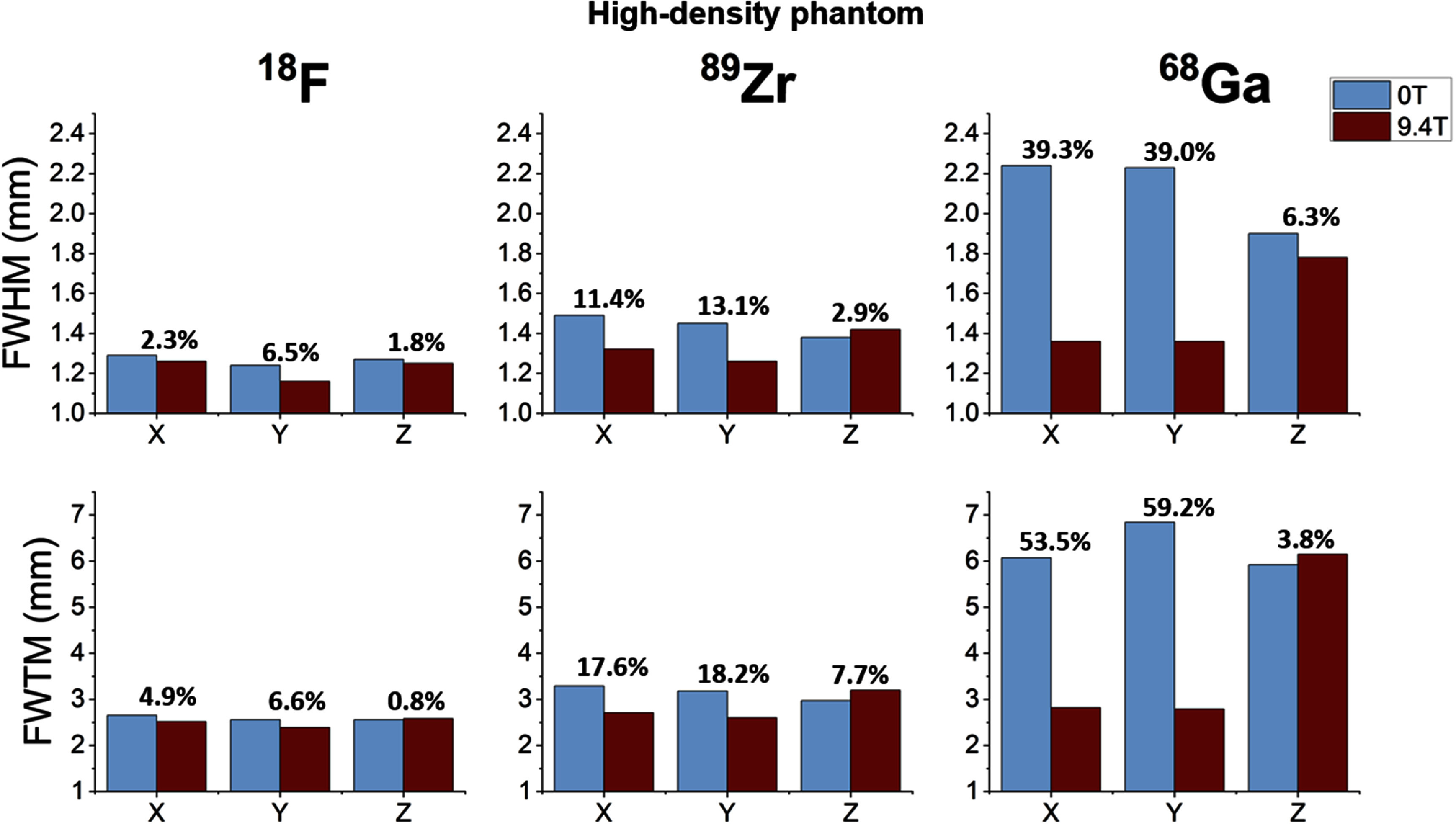
FWHM (top row) and FWTM (bottom row) obtained from the capillary profiles for high-density phantom. For each isotope (^18^F, ⁸⁹Zr, ^6^⁸Ga), the metrics are shown along the three spatial directions (x, y, z) and for the two MRI field conditions (0 T in blue and 9.4 T in red). The percentage numbers above the bars indicate the relative change between 9.4 T and 0 T. Error bars represent the standard deviation across the seven analyzed slices.

Following this analysis strategy, the quantitative results derived for all isotopes, materials and spatial directions are summarized in figures 5–7. for the low-, medium- and high-density phantoms, respectively. In each figure, the FWHM and FWTM values obtained at 0 T and 9.4 T are shown for the three spatial directions, together with the relative percentage difference between the two field conditions, computed with respect to the 0 T measurements.

Across all acquisition conditions, the low-density phantom showed very similar behavior for the three isotopes (see figure 5). The FWHM values remained within 1.16–1.38 mm for all directions, and the FWTM values within 2.20–2.74 mm. The relative differences between the 0 T and 9.4 T measurements were below 5.8% for the FWHM and below 8.1% for the FWTM.

In contrast to the low-density phantom, the results obtained for the medium- and high- density phantoms (see figures 6 and 7) revealed clear differences between isotopes and a stronger dependence on the surrounding material. For both phantoms, the profiles of ^18^F remained narrow and largely unchanged across all directions, with FWHM values around 1.2–1.3 mm and FWTM values between 2.3 and 2.6 mm, corresponding to relative differences below 3.1% and 7.6% for the medium-density phantom and below 6.5% and 6.6% for the high-density phantom, for the FWHM and FWTM, respectively. The results for ⁸⁹Zr followed the same general trend, showing only a moderate broadening relative to ^18^F and limited sensitivity to the magnetic field, with relative differences not exceeding 9.0% and 14.0% in the medium-density phantom and 13.1% and 18.2% in the high-density phantom, for the FWHM and FWTM, respectively.

For ^6^⁸Ga, substantially broader distributions were observed outside the magnetic field, with FWTM values between 6 and 7 mm in both phantoms. When the acquisitions were performed inside the 9.4 T MRI system, a marked transverse confinement was observed, with relative reductions of 32.5% (medium-density) and 39.3% (high-density) in the FWHM, and of 59.8% (medium-density) and 59.2% (high-density) in the FWTM, whereas the axial direction remained essentially unchanged.

### 3.2. MicroDerenzo

**Figure 8.**
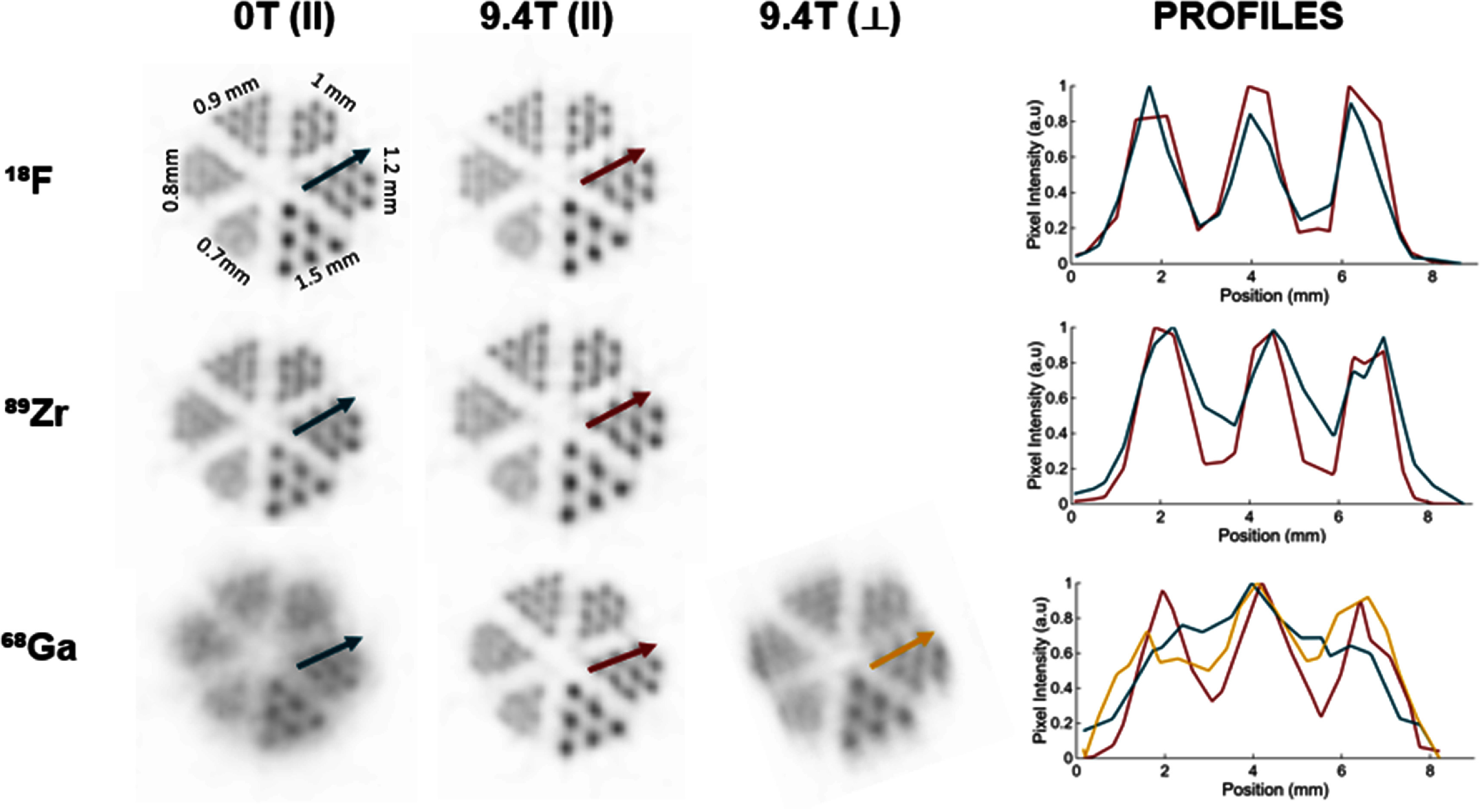
Reconstructed images of the microDerenzo phantom for ^18^F, ⁸⁹Zr and ^6^⁸Ga acquired at 0 T and at 9.4 T in both axial and rotated configurations. Line profiles from the 1.2 mm rod sector are shown on the right.

The microDerenzo evaluation is depicted in figure 8 and summarized in table 3. In this figure one can qualitatively observe that the two ^18^F cases (0 T and 9.4 T) do not differ discernably. The images for ⁸⁹Zr show some visual enhancement when measuring in the 9.4 T field, and the enhancement is significantly improved for the case of ^6^⁸Ga. Even when the rods of the ^6^⁸Ga case are perpendicular to the magnetic field orientation, a partial improvement in rod resolvability is observed.

**Table 3.** Valley-to-peak ratio (VPR) values for each rod sector of the microDerenzo phantom under the three acquisition conditions: 0 T, 9.4 T, and 9.4 T with the phantom rotated 90° (┴).

		Rod size (mm)					
Isotope	B_0_	1.5	1.2	1.0	0.9	0.8	0.7
^18^F	0 T (||)	**0.200**	**0.469**	**0.609**	**0.668**	0.927	0.941
	9.4 T(||)	**0.161**	**0.379**	**0.388**	**0.619**	0.922	0.942

⁸⁹Zr	0 T (||)	**0.385**	**0.670**	0.782	0.815	0.968	0.977
	9.4 T (||)	**0.188**	**0.395**	**0.518**	**0.644**	0.922	0.950

^6^⁸Ga	0 T (||)	0.739	0.970	0.976	0.941	0.975	0.978
	9.4 T (||)	**0.290**	**0.566**	0.752	0.803	0.962	0.975
	9.4 T (┴)	**0.659**	**0.729**	0.886	0.945	0.937	0.973

*Note:* Bold values indicate sectors where the average VPR < 0.735

Table 3 reports the VPR obtained for each rod sector under the different PET/MRI conditions. The most pronounced changes occur for ^6^⁸Ga, whose higher positron range leads to substantial blurring in the absence of a magnetic field. At 0 T, the Rayleigh criterion is not satisfied for any rod size, even for the largest rods (1.5 mm). Within a 9.4 T field, however, resolvability improves notably, allowing to almost resolve the 1.0 mm sector. When the phantom is rotated by 90° with respect to *B*_0_, this enhancement becomes orientation-dependent, again almost resolving the 1.0 mm rods sector.

**Figure 9.**
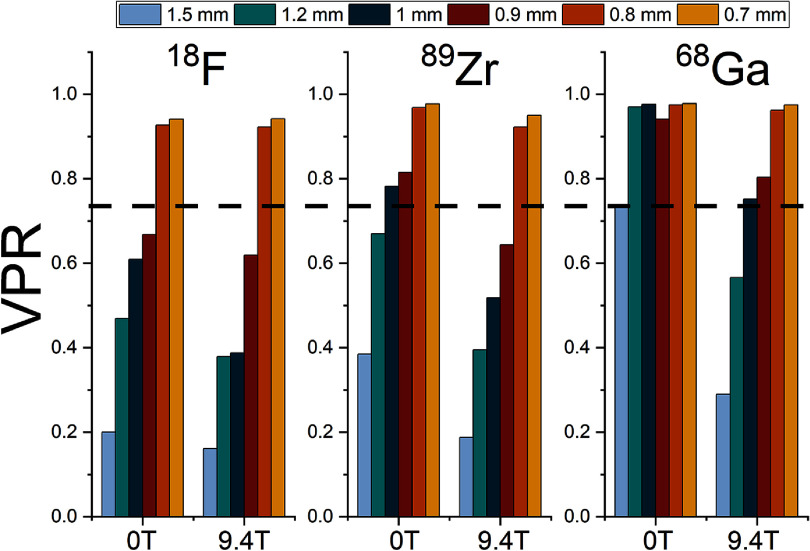
VPR for each rod diameter sector of the microDerenzo phantom (II B_0_), shown for the three isotopes (^18^F, ⁸⁹Zr, ^6^⁸Ga) under the 0 T and 9.4 T conditions. The dashed line indicates the Rayleigh threshold (VPR = 0.735).

To provide a clearer visual comparison across isotopes, rod sizes and magnetic-field conditions, figure 9 displays these VPR values in graphical form for measurements performed with the microDerenzo phantom aligned with *B*_0_. Consistently with the qualitative inspection, for ^18^F, the VPR values show that the magnetic field had negligible impact on rod detectability: in both the 0 T and 9.4 T acquisitions, rods as small as to 0.9 mm remained below the Rayleigh threshold. In contrast to this, for the ⁸⁹Zr case, the VPR results confirm an improvement in the presence of the magnetic field. At 0 T only rods from 1.2 mm in diameter were resolvable, but when measuring in the 9.4 T field the 0.9 mm rods have VPR values below the threshold as well.

## 4. Discusion

The capillary measurements provide a detailed characterization of how positron-range changes as a function of isotope energy, material (tissue) density, and magnetic field influence.

In the low-density phantom (see figure 5), all three isotopes produced very similar profiles, with FWHM and FWTM values remaining within 1.2–1.4 mm and FWTM values within 2.2–2.7 mm, independent of the magnetic field. This behavior is consistent with the hypothesis that, in very low-density surrounding media, the densities of the water in the 18-F solution (∼1.0 g cm^−3^) in the capillaries and the glass capillary walls (∼2.2 g cm^−3^) exceed that of the lung-equivalent medium (∼0.2 g cm^−3^). Accordingly, for low energy positron emitters (e.g. ^18^F) a large fraction of positrons annihilate inside the capillaries, and an appreciable fraction of those escaping the capillaries also escape the phantom before annihilation. This causes the measured profiles to be dominated by the geometry of the capillary rather than by the properties of the surrounding material (Kemerink *et al* 2011). When using medium- and high-density phantoms (see figures 6 and 7), the larger stopping power (i.e. the higher energy loss per unit path length experienced by the positrons in the medium) allows the differences between isotopes and magnetic field conditions to emerge. For ^18^F and ⁸⁹Zr, the changes observed from the introduction of the magnetic field were small, with FWHM values around 1.2–1.5 mm and FWTM values remaining within 2.3–3.4 mm for both materials and fields, consistent with their relatively low positron emission energies. The situation differed for ^6^⁸Ga, whose higher positron energy produced a substantially larger fraction of annihilations to occur outside of the capillaries in both phantoms. In the presence of the 9.4 T magnetic field, the magnetic Lorenz force produced a measurable reduction in transverse positron range, particularly observable in the medium- and high-density phantoms: the FWHM decreased by approximately 32% in medium-density phantom and 39% in the high-density phantom, while the transverse FWTM was reduced by about 58% and 56%, respectively. Nevertheless, the annihilation distribution in the axial direction remained essentially unchanged.

These results align well with previous simulation studies reporting an energy-dependent transverse confinement of the positron range for high-energy emitters (Soultanidis *et al* 2011, Li *et al* 2017), as well as with experimental data from clinical and preclinical PET/MR systems, where improvements were mainly observed for medium- and high-energy isotopes (Huang *et al* 2014, Shah *et al* 2014, Pollard *et al* 2022). In particular, the magnitude of the changes in transverse spatial extent of annihilation sites measured for ^6^⁸Ga in the medium- and high-density phantoms in this study are consistent with the confinement patterns described by Carter et al (2020) and Ku-Toval et al (2024), both reporting a significant reduction of the transverse range with minimal changes along the direction parallel to the magnetic field.

However, the impact of phantom physical density on transverse annihilation distribution as reported in this work appears to disagree in some aspects with those of some other published results: previous studies report reductions in transverse positron range with higher material density because of their higher electron density (Alva-Sánchez *et al* 2016, Ku-Toval *et al* 2024); however, in our measurements the FWHM and FWTM of the annihilation profiles increase with increasing material density. This can be explained by the geometry and scale of our experiment, and the metrics used to quantify the annihilation profile characteristics. For this study, the phantoms were designed with dimensions comparable to those of small-animal organs (for example, mice lungs extend approximately 10–12 mm only in its largest transverse axis). Therefore, for ^6^⁸Ga in the low-density phantom, a substantial fraction of positrons escapes the phantom before annihilation. As a result, the measured profiles become dominated by intra-capillary annihilations rather than by true phantom-specific density modulated trajectories in the surrounding material, preventing the identification of density-dependent positron range effects that are otherwise visible in the medium- and high-density phantoms.

The microDerenzo measurements support the trend observed for the capillary experiments, but for more complex structures and under a surrounding material density comparable to that of the medium-density capillary phantom. Figure 9 shows that for ^18^F, rod sizes down to 0.9 mm were resolvable at both 0 T and 9.4 T. For ⁸⁹Zr, rods of only 1.2 mm diameter were resolvable at 0 T, while rods of 0.9 mm also satisfied the Rayleigh criterion at 9.4 T, consistent with the modest narrowing observed in the FWTM values. The most significant changes occurred for ^6^⁸Ga: at 0 T only the 1.5 mm rods were resolvable, whereas at 9.4 T the VPR for the 1.0 mm sector fell just slightly above the Rayleigh detectability threshold. When the phantom is oriented with the rods perpendicular to B_0_, only one of the two in-plane directions of the rods is affected by the magnetic field confinement, but this partial confinement is still sufficient to further reduce the VPR values for the 1.2 mm rods compared to 0 T, resulting in a measurable improvement in spatial resolution.

Overall, there was an agreement between the capillary measurements and the microDerenzo. The capillary profiles provide a sensitive and controlled way to characterize positron-range effects across different materials and magnetic field conditions. These findings demonstrate that strong magnetic fields can reduce the blurring associated with high-energy positrons in preclinical PET systems, particularly in tissue-equivalent materials. Several limitations should be considered when interpreting these results: the phantoms used here are homogeneous and simplified compared to *in-vivo* environments and their dimensions may limit the fraction of positrons that annihilate within the material, especially in low-density mediums. It should be noted that no attenuation or scatter corrections were applied in this study; therefore, comparisons of profile widths across different phantom materials should be interpreted with caution, as density-dependent attenuation and scatter may influence count statistics and the profile tails. However, for each phantom material, the comparison between measurements acquired inside and outside the magnetic field was performed using the same phantom geometry and composition, so attenuation-related effects were canceled out. Consequently, the observed differences between the 0 T and 9.4 T measurements for a given material can be attributed to magnetic-field-induced positron range confinement.

Despite these limitations, the combined behavior observed in the different density phantoms offers a well-controlled framework for understanding how isotope energy, material density and magnetic field interact, and provides experimental evidence that can help guide future PET/MRI studies in more complex and heterogeneous scenarios. Beyond these observations, the present study provides, to our knowledge, the first systematic experimental characterization of positron range confinement across multiple isotopes and tissue-equivalent materials in a high-field using a preclinical PET system, highlighting why this information is essential for interpreting PET/MRI images and understanding its impact on PET spatial resolution.

## 5. Conclusions

This study investigates the effect of a strong static magnetic field (9.4 T) on positron range confinement and its impact on PET spatial resolution in tissue-equivalent materials. The results show a clear reduction of transverse blurring for ^6^⁸Ga in the plane perpendicular to *B*_0_, while the image resolution in the axial direction remains essentially unchanged. However, much smaller changes in overall spatial resolution were observed for ^18^F and ⁸⁹Zr. The effect on positron range of background material density is negligible in low-density phantoms but becomes pronounced in medium- and high-density capillary phantoms. The microDerenzo measurements further demonstrate the efficacy of magnetic positron confinement for isotopes with high positron emission energies by showing improved rod resolvability for ^6^⁸Ga under the 9.4 T field.

Overall, the results highlight the relevance of studies of magnetic field strength and tissue-equivalent density as they affect PET/MRI images, especially those obtained with high-energy isotopes. Future work should extend this analysis to *in-vivo* studies, heterogeneous mediums, and reconstruction approaches that incorporate the anisotropic behavior of positron-range suppression, as well as to dedicated quantitative evaluations aimed at assessing its impact on the images. As an additional line of investigation, measurements performed at different magnetic-field strengths would help determine how strongly the observed effects depend on field intensity and whether intermediate values already provide a significant benefit for medium- and high-energy emitters.

## Data Availability

The data that support the findings of this study are available from the corresponding author upon reasonable request. The data are not publicly available at the time of publication due to their large size and the absence of an institutional repository for this dataset.

## References

[pmbae6d7cbib1] Alva-Sánchez H, Quintana-Bautista C, Martínez-Dávalos A, Ávila-Rodríguez M A, Rodríguez-Villafuerte M (2016). Positron range in tissue-equivalent materials: experimental microPET studies. Phys. Med. Biol..

[pmbae6d7cbib2] Bettinardi V, Castiglioni I, De Bernardi E, Gilardi M C (2014). PET quantification: strategies for partial volume correction. Clin. Transl. Imaging.

[pmbae6d7cbib3] Bruker Corporation (2024). BioSpec 94/20 USR MRI scanner specifications. https://www.bruker.com/en/products-and-solutions/preclinical-imaging/mri/biospec/biospec-70-20-and-94-20.html.

[pmbae6d7cbib4] Cal-González J, Herraiz J L, España S, Corzo P G, Vaquero J J, Desco M, Udias J M (2013). Positron range estimations with PeneloPET. Phys. Med. Biol..

[pmbae6d7cbib5] Carter L M, Kesner A L, Pratt E C, Sanders V A, Massicano A V F, Cutler C S, Lewis J S, Lewis J S (2020). The impact of positron range on PET resolution, evaluated with phantoms and PHITS monte carlo simulations for conventional and non-conventional radionuclides. Mol. Imaging Biol..

[pmbae6d7cbib6] Chen W, Cloughesy T, Kamdar N, Satyamurthy N, Bergsneider M, Liau L, Silverman D H, Czernin J, Phelps M E, Silverman D H S (2005). Imaging proliferation in brain tumors with ^18^F-FLT PET: comparison with ^18^F-FDG. J. Nucl. Med..

[pmbae6d7cbib7] Cheng S (2018). Positron emission tomography imaging of prostate cancer with ^6^⁸Ga-labeled gastrin-releasing peptide receptor agonist BBN7–14 and antagonist RM26. Bioconjug. Chem..

[pmbae6d7cbib8] Daou D (2008). Respiratory motion handling is mandatory to accomplish the high-resolution PET destiny. Eur. J. Nucl. Med. Mol. Imaging.

[pmbae6d7cbib9] Dijkers E C, Oude Munnink T H, Kosterink J G, Brouwers A H, Jager P L, De Jong J R, De Vries E G, Schröder C P, Lub-de Hooge M N, de Vries E G (2010). Biodistribution of ⁸⁹Zr-trastuzumab and PET imaging of HER2-positive lesions in patients with metastatic breast cancer. Clin. Pharmacol. Ther..

[pmbae6d7cbib10] Ehman E C, Johnson G B, Villanueva-Meyer J E, Cha S, Leynes A P, Larson P E Z, Hope T A (2017). PET/MRI: where might it replace PET/CT?. J. Magn. Reson. Imaging.

[pmbae6d7cbib11] Finnfoam S L (2025). EPS 100 foam insulation technical data sheet. https://finnfoam.es/wp-content/uploads/2025/03/Ficha-tecnica-EPS-100-ESP-2025.pdf.

[pmbae6d7cbib12] Formlabs (2022). Rigid 10K Resin Technical Data Sheet (FLRG1001), Document 2001479-TDS-ENUS-0. https://formlabs-media.formlabs.com/datasheets/2001479-TDS-ENUS-0.pdf.

[pmbae6d7cbib13] Freire M, Gonzalez-Montoro A, Sanchez F, Benlloch J M, Gonzalez A J (2019). Calibration of gamma-ray impacts in monolithic-based detectors using voronoi diagrams. IEEE Trans. Radiat. Plasma Med. Sci.

[pmbae6d7cbib14] González A J (2016). The MINDView brain PET detector, feasibility study based on SiPM arrays. Nucl. Instrum. Methods Phys. Res. A.

[pmbae6d7cbib15] Gonzalez A J (2018). Initial results of the MINDView PET insert inside the 3 T mMR. IEEE Trans. Radiat. Plasma Med. Sci..

[pmbae6d7cbib16] Gsell W (2020). Characterization of a preclinical PET insert in a 7 tesla MRI scanner: beyond NEMA testing. Phys. Med. Biol..

[pmbae6d7cbib17] Hallen P, Schug D, Schulz V (2020). Comments on the NEMA NU 4–2008 standard on performance measurement of small animal positron emission tomographs. EJNMMI Phys..

[pmbae6d7cbib18] Hammer B E, Christensen N L, Heil B G (1994). Use of a magnetic field to increase the spatial resolution of positron emission tomography. Med. Phys..

[pmbae6d7cbib19] Herzog H, Van Den Hoff J (2012). Combined PET/MR systems: an overview and comparison of currently available options. Q. J. Nucl. Med. Mol. Imaging.

[pmbae6d7cbib20] Hofmann M, Steinke F, Scheel V, Charpiat G, Farquhar J, Aschoff P, Pichler B J, Schölkopf B, Pichler B J (2008). MRI-based attenuation correction for PET/MRI: a novel approach combining pattern recognition and atlas registration. J. Nucl. Med..

[pmbae6d7cbib21] Huang S Y, Savic D, Yang J, Shrestha U, Seo Y (2014). The effect of magnetic field on positron range and spatial resolution in an integrated whole-body time-of-flight PET/MRI system.

[pmbae6d7cbib22] James M L, Gambhir S S (2012). A molecular imaging primer: modalities, imaging agents, and applications. Physiol. Rev..

[pmbae6d7cbib23] Judenhofer M S, Cherry S R (2013). Applications for preclinical PET/MRI. Semin. Nucl. Med..

[pmbae6d7cbib24] Kemerink G J, Visser M G, Franssen R, Beijer E, Zamburlini M, Halders S G, Teule G, Mottaghy F M, Teule G J J (2011). J 2011 Effect of the positron range of ^18^F, ^68^Ga and 12^4^I on PET/CT in lung-equivalent materials. Eur. J. Nucl. Med. Mol. Imaging.

[pmbae6d7cbib25] Kraus R, Delso G, Ziegler S I (2012). Simulation study of tissue-specific positron range correction for the new Biograph mMR whole-body PET/MR system. IEEE Trans. Nucl. Sci..

[pmbae6d7cbib26] Ku-Toval D, Rodríguez-Villafuerte M, Ávila-Rodríguez M A, Martinez-Davalos A, Schalch J M, Alva-Sánchez H (2024). Quantitative analysis of the effect of the magnetic field generated by a PET/MR scanner on positron range. Phys. Med. Biol..

[pmbae6d7cbib27] Li C, Cao X, Liu F, Tang H, Zhang Z, Wang B, Wei L (2017). Compressive effect of the magnetic field on the positron range in commonly used positron emitters simulated using Geant4. Eur. Phys. J. Plus.

[pmbae6d7cbib28] Li Y, Zhao H, Hu S, Zhang X, Chen H, Zheng Q (2023). PET imaging with [^6^⁸Ga]-labeled TGFβ-targeting peptide in a mouse PANC-1 tumor model. Front. Oncol..

[pmbae6d7cbib29] Lopez-Berenguer F, Gonzalez-Montoro A, Freire M, González-Tamarit A, Jiménez-Serrano S, Vidal L F, González A J (2025). Evaluation of a PET insert for trimodal imaging: a step towards PET/MRI-guided focused ultrasound. IEEE Trans. Radiat. Plasma Med. Sci..

[pmbae6d7cbib30] Luo Z, Gao X, Pu S, Ding K, Zhang X (2025). Development of a ^6^⁸Ga-labeled peptide tracer targeting TREM2 for neuroinflammation PET imaging. J. Nucl. Med..

[pmbae6d7cbib31] Mannheim J G, Schmid A M, Schwenck J, Katiyar P, Herfert K, Pichler B J, Disselhorst J A (2018). PET/MRI hybrid systems. Semin. Nucl. Med..

[pmbae6d7cbib32] Moses W W (2011). Fundamental limits of spatial resolution in PET. Nucl. Instrum. Methods Phys. Res. A.

[pmbae6d7cbib33] Nensa F, Kloth J, Tezgah E, Poeppel T D, Heusch P, Goebel J, Schlosser T, Schlosser T (2018). Feasibility of FDG-PET in myocarditis: comparison to CMR using integrated PET/MRI. J. Nucl. Cardiol..

[pmbae6d7cbib34] Ory D (2015). PET imaging of TSPO in a rat model of local neuroinflammation induced by intracerebral injection of lipopolysaccharide. Nucl. Med. Biol..

[pmbae6d7cbib35] Phelps M E, Hoffman E J, Huang S C, Ter-Pogossian M M (1975). Effect of positron range on spatial resolution. J. Nucl. Med..

[pmbae6d7cbib36] Pollard A C, de la Cerda J, Schuler F W, Kingsley C V, Gammon S T, Pagel M D (2022). Evaluations of the performances of PET and MRI in a simultaneous PET/MRI instrument for pre-clinical imaging. EJNMMI Phys..

[pmbae6d7cbib37] PRO (2025). 1.75 mm White PLA 3D printer filament data sheet. https://docs.rs-online.com/be57/A700000007511193.pdf.

[pmbae6d7cbib38] Shah N J, Herzog H, Weirich C, Tellmann L, Kaffanke J, Caldeira L, Iida H, Qaim S M, Coenen H H, Iida H (2014). Effects of magnetic fields of up to 9.4 T on resolution and contrast of PET images as measured with an MR-BrainPET. PLoS One.

[pmbae6d7cbib39] Shao Y, Cherry S R, Farahani K, Meadors K, Siegel S, Silverman R W, Marsden P K (1997). Simultaneous PET and MR imaging. Phys. Med. Biol..

[pmbae6d7cbib40] Shepp L A, Vardi Y (1982). Maximum likelihood reconstruction for emission tomography. IEEE Trans. Med. Imaging.

[pmbae6d7cbib41] Soret M, Bacharach S L, Buvat I (2007). Partial-volume effect in PET tumor imaging. J. Nucl. Med..

[pmbae6d7cbib42] Soultanidis G, Karakatsanis N, Nikiforidis G, Loudos G (2011). Study of the effect of magnetic field in positron range using GATE simulation toolkit. J. Phys.: Conf. Ser..

[pmbae6d7cbib43] Toussaint M, Loignon-Houle F (2023). Evaluation of spatial resolvability in hot spot phantoms (Version 1.0.1) [Computer software]. https://github.com/MaxTousss/PetSpatialResolvability.

[pmbae6d7cbib44] Van Dalen J (2008). Effect of the positron range on the spatial resolution of a new generation preclinical PET scanner using ^18^F, ^6^⁸Ga, ⁸⁹Zr and ^12^⁴I. Eur. J. Nucl. Med. Mol. Imaging.

[pmbae6d7cbib45] Wirrwar A, Vosberg H, Herzog H, Halling H, Weber S, Müller-Gärtner H W (1997). Tesla magnetic field reduces range of high-energy positrons: potential implications for positron emission tomography. IEEE Trans. Nucl. Sci..

